# Four-Photon Molecular Upconversion

**DOI:** 10.1021/jacs.6c17238

**Published:** 2026-10-02

**Authors:** Seamus S. Lowe, Maxwell Sun, Jesse A. Wisch, Kostubh Gaur, Chiao-Jung Su, Azka Arshad, Felix N. Castellano, John E. Anthony, Noel C. Giebink, Barry P. Rand

**Affiliations:** † Department of Mechanical & Aerospace, Engineering, Princeton University, Princeton, New Jersey 08544, United States; ‡ Department of Electrical Engineering and Computer Science, 1259 University of Michigan, Ann Arbor, Michigan 48109, United States; § Department of Electrical and Computer Engineering, 6740 Princeton University, Princeton, New Jersey 08544, United States; ∥ Department of Chemistry, 4530 University of Kentucky, Lexington, Kentucky 40506, United States; ⊥ Department of Chemistry, 6798 North Carolina State University, Raleigh, North Carolina 27695, United States; # Andlinger Center for Energy and the Environment, Princeton University, Princeton, New Jersey 08544, United States

## Abstract

Triplet-triplet annihilation (TTA) upconversion converts low-energy photons into higher-energy emission, but conventional TTA is limited by its two-photon nature, as the emitted photon energy cannot exceed twice the excitation photon energy. Here, a fully organic solid-state thin-film architecture cascades two TTA upconversion stages through an intermediate Förster resonance energy transfer (FRET) bridge to achieve molecular four-photon upconversion. The first sensitizer/annihilator bilayer upconverts near-infrared (NIR) excitation into intermediate singlets, which are transferred through the FRET bridge to the sensitizer of the second TTA stage. The second sensitizer/annihilator bilayer performs a second TTA step to generate blue emission. The device converts 904 nm excitation into 445 nm emission, corresponding to a 1.41 eV anti-Stokes shift and an emitted photon energy 2.03 times the excitation photon energy, exceeding the maximum possible energy gain with conventional two-photon TTA. Because two quadratic TTA stages are coupled in series, the ideal low-intensity response of the four-photon device is quartic when both stages operate below threshold, providing a steeper nonlinearity desirable for nonlinear sensing. Experimentally, power-dependent measurements reveal a superquadratic slope of 3.03, followed by quadratic and linear regimes as the constituent TTA stages cross their thresholds. Independent stage measurements and time-resolved photoluminescence dynamics support the four-photon mechanism.

## Graphical abstract

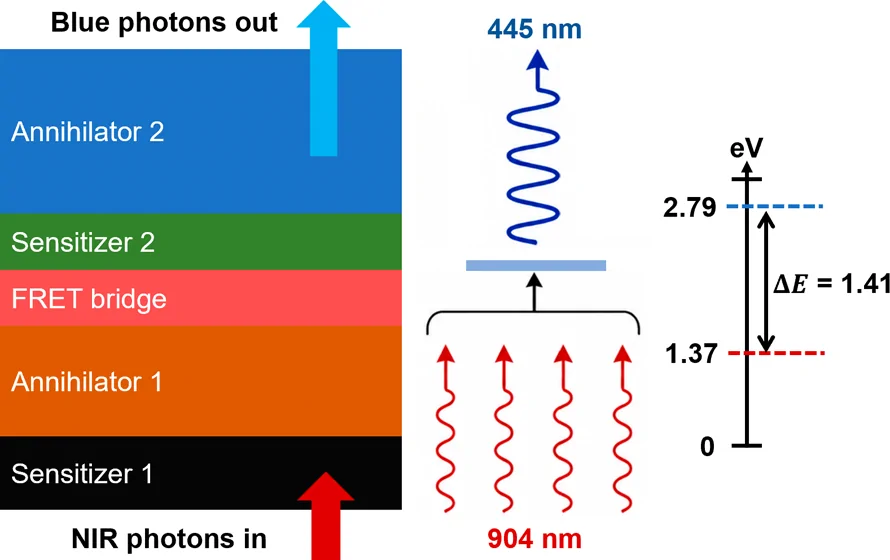

## 1 Introduction

Photon upconversion, the conversion of low-energy photons into higher-energy excited states or emitted photons, allows for the harvesting of low-energy photons to drive higher-energy processes. In solar energy harvesting, upconversion allows utilization of sub-bandgap photons. In photoredox catalysis and photochemistry, upconversion can enable high-energy reactions using lower-energy excitation, which may be more abundant and readily available. In bioimaging and optical manufacturing, upconversion can convert deeply penetrating near-infrared (NIR) light into shorter-wavelength optical responses.^1–10^ Triplet-triplet annihilation (TTA) upconversion, also known as triplet-fusion upconversion, is a widely developed upconversion mechanism which typically utilizes tightly bound excitons in molecular systems.^2–6^ In a conventional TTA system, a sensitizer material absorbs low-energy photons, populating singlet excitons in the sensitizer (Figure 1a). Most commonly, these singlets are then converted into triplet excitons via intersystem crossing (ISC) and then transfer to triplets in the annihilator material through Dexter energy transfer (DET), a particular type of triplet energy transfer (TET), as depicted in Figure 1c. Two annihilator triplets then undergo TTA to generate one high-energy singlet exciton that emits an upconverted photon. This bimolecular excitonic mechanism gives TTA upconversion its characteristic nonlinear power dependence. At low excitation intensities, the upconversion emission intensity exhibits a quadratic dependence on intensity. After the threshold intensity, *I*_*th*_, the power dependence transitions into a linear regime as upconversion switches from a regime dominated by first-order triplet decay to one in which bimolecular TTA becomes efficient.^3–6,11,12^

**Figure 1:**
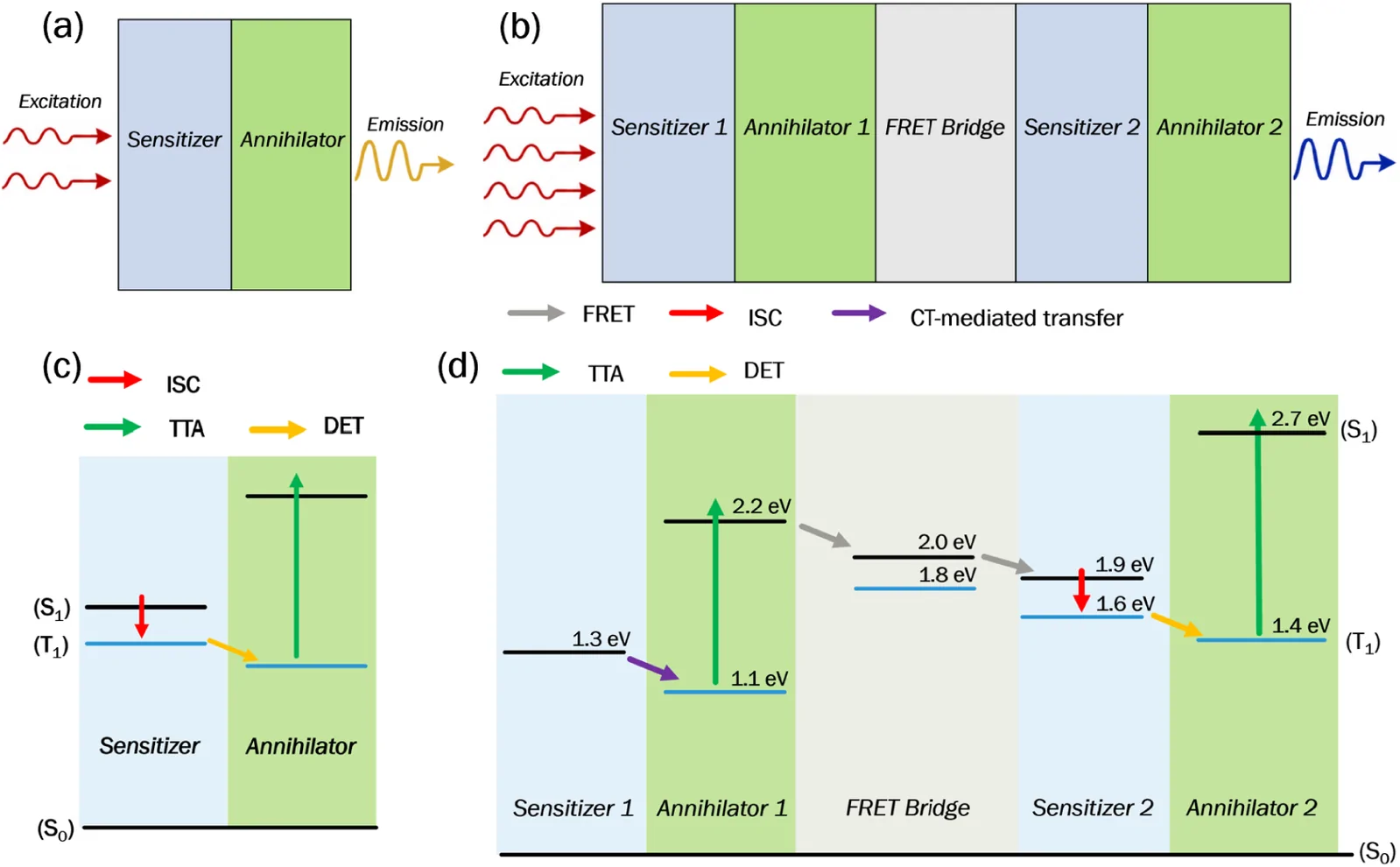
(a) Conventional two-photon TTA upconversion bilayer. (b) Thin-film device structure for cascaded energy transfer for four-photon upconversion. (c) Jablonski diagram of conventional two-photon TTA upconversion. (d) Jablonski diagram for four-photon upconversion including energy levels corresponding to molecules introduced in Figure 2.

Despite these advantages, the photon energy gain, or anti-Stokes shift, achievable by standard TTA upconversion is fundamentally constrained by its two-photon nature. As one high-energy singlet is produced from the energy of two triplet excitons generated by two absorbed photons, the emitted photon energy cannot exceed twice the excitation photon energy, even before accounting for energetic losses during ISC, TET, and TTA.^2–6,13^ In practice, these losses make large anti-Stokes shifts difficult to achieve, particularly in solid-state systems where triplet diffusion is limited.^7,8,14^ Considerable progress has been made in improving conventional solid-state TTA upconversion through the use of polymer hosts, molecular glasses, crystalline organic semiconductors, nanocrystal and 2D sensitizers, plasmonic enhancement, and interface-mediated triplet generation.^7,8,14–19^ However, these systems remain constrained by the conventional two-photon TTA upconversion framework, and therefore cannot access emitted photon energies greater than twice the excitation photon energy. Additionally, while conventional two-photon TTA upconversion exhibits quadratic optical nonlinearity, nonlinear upconversion responses can provide enhanced spatial confinement and resolution in applications including optical thresholding, high-contrast imaging, background-free detection, nonlinear optical sensing, and additive manufacturing.^20–22^ Higher orders of nonlinearity can therefore provide an even steeper intensity-dependent response.

In this work, we propose and demonstrate a fully organic solid-state thin-film device architecture to surpass this limitation by using four lower-energy photons to generate one higher-energy photon, or four-photon upconversion. This is achieved by cascaded energy transfer through multiple upconversion stages, mediated by a Förster resonance energy transfer (FRET) bridge such that the output of one nonlinear TTA stage serves as the pump for the next nonlinear TTA stage (Figure 1b). To realize this process, the sensitizer in the first sensitizer/annihilator bilayer stage absorbs the incident NIR excitation and sensitizes triplets in an annihilator which undergo TTA to produce the first set of upconverted singlets in the red/yellow energy range. Downhill FRET of these intermediate singlets to singlets in the second sensitizer is mediated by a thin film with an intermediate singlet energy. The intermediate layer acts as a FRET bridge, accepting singlets from the first annihilator and transferring them to the sensitizer of the second sensitizer/annihilator bilayer stage. The second sensitizer then sensitizes triplets in the second annihilator, where a second TTA event generates the final blue-emitting singlet excitons. This process is shown in Figure 1d. In this way, two sequential two-photon TTA processes are coupled into a four-photon upconversion process. As depicted in Figure 1d, the first upconversion stage utilizes a different TET mechanism where, as opposed to the typical ISC and DET process, sensitizer excitons transfer to annihilator triplets through a charge-transfer-mediated (CT-mediated) mechanism.

The resulting four-photon upconversion device produces blue emission at 445 nm under NIR excitation as long as 904 nm. This gives a maximum anti-Stokes shift of 1.41 eV which is greater than the excitation photon energy. The emitted photon energy is, therefore, 2.03× the excitation photon energy, exceeding the maximum theoretical energy gain of a conventional two-photon TTA upconversion process. This result establishes that cascaded solid-state TTA devices can access anti-Stokes shifts which are impossible with conventional TTA upconversion. Additionally, as the first nonlinear TTA stage pumps the second nonlinear TTA stage, the demonstrated device should exhibit a quartic power-dependence regime when both stages are below threshold, a quadratic regime when one stage is above threshold, and a linear regime when both stages are above threshold. We observe a logarithmic slope of 3.03 at the lowest measured excitation intensities, demonstrating a superquadratic optical response and providing a steeper optical nonlinearity than can be achieved with a conventional TTA upconversion system. Power-dependent and time-resolved photoluminescence (TRPL) measurements of the independent sensitizer/annihilator bilayer stages further verify the mechanism of the cascaded upconversion process by revealing delayed TTA emission dynamics from each annihilator. These measurements confirm that both bilayers function as independent solid-state TTA upconversion stages, providing the mechanistic basis for coupling their nonlinear responses into the observed four-photon upconversion process.

## 2 Results and Discussion

The four-photon upconversion architecture requires two independent TTA upconversion stages that can be coupled through an intermediate FRET step. The first stage must absorb NIR photons and generate an intermediate upconverted singlet exciton, while the second stage must accept this intermediate singlet and perform a second TTA process to generate the final blue-emitting singlet. A thin FRET bridge between the TTA stages allows for downhill energy transfer from the first annihilator to the second sensitizer while suppressing back transfer of triplets in the second sensitizer to triplet states in the first annihilator. To satisfy these requirements, Y6, also known as BTP-4F, was selected as the NIR absorbing first-stage sensitizer, rubrene as the first-stage annihilator, DCM (4-(dicyanomethylene)-2-methyl-6-(4-dimethylaminostyryl)-4H-pyran) as the FRET bridge, PtTPTBP (platinum (II) tetraphenyltetrabenzoporphyrin) as the second-stage sensitizer, and TIPS-ANT (9,10-bis­[(triisopropylsilyl)­ethynyl]­anthracene) as the second-stage annihilator (molecular structures and energy flow shown in Figure 2a). The approximately 20 nm Y6 film provides strong NIR absorption, as shown in Figure 2c, as low as 904 nm and sensitizes triplets in the neighboring 10 nm rubrene annihilator film through charge transfer (CT)-mediated energy transfer.^19^ In the rubrene film, TTA produces rubrene singlets, which exhibit spectral overlap with the absorption of DCM as shown in Figure 2b, allowing for downhill FRET to singlets in the neighboring 4 nm DCM film. Singlets in DCM, in turn, exhibit strong spectral overlap with the red absorption band of PtTPTBP as shown in Figure 2d, enabling downhill FRET to singlets in the neighboring 2 nm PtTPTBP film. Singlets in PtTPTBP then undergo intersystem crossing (ISC) to triplets which transfer to TIPS-ANT via Dexter energy transfer (DET). The DCM film is designed to suppress back transfer of triplets in PtTPTBP to lower-energy triplet states in rubrene. As shown in Figure 1d, the triplet energy of DCM is greater than that of PtTPTBP and substantially greater than that of rubrene, making triplet transfer from PtTPTBP to DCM energetically uphill. The DCM layer also spatially separates PtTPTBP from rubrene and is therefore expected to suppress DET back transfer that would compete with the upconversion cascade. Finally, triplets in the 60 nm TIPS-ANT film undergo TTA to produce the final high-energy singlets which yield the high-energy blue emission up to 445 nm as shown in Figure 2c.

**Figure 2:**
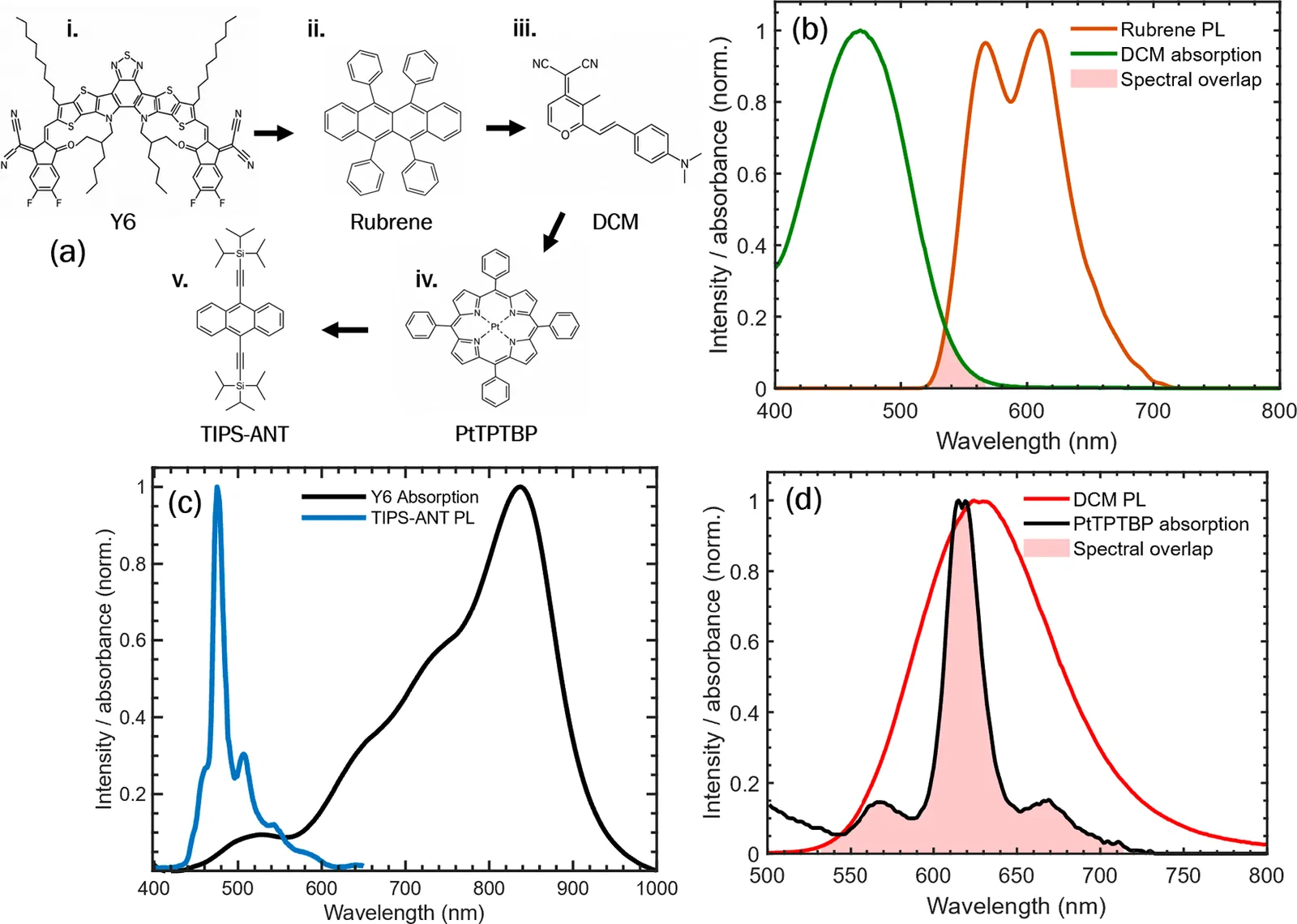
(a) Molecular energy cascade roadmap with molecular structures for i. Y6, ii. rubrene, iii. DCM, iv. PtTPTBP, and v. TIPS-ANT. (b) Spectral overlap of rubrene PL and DCM UV-Vis absorption. (c) Y6 UV-Vis absorption of incoming NIR excitation and TIPS-ANT photoluminescence (PL) producing blue anti-Stokes emission. (d) Spectral overlap of DCM PL and PtTPTBP UV-Vis absorption.

The individual layer thicknesses were selected to balance the competing requirements of absorption, excitonic transport, and energy transfer through the device. The ∼20 nm Y6 film provides substantial NIR absorption while keeping photoexcited states within efficient exciton diffusion distance to the Y6/rubrene interface. The 10 nm rubrene film provides sufficient volume for triplet transport and TTA while maintaining efficient FRET coupling of rubrene singlets to the adjacent DCM layer. The 4 nm DCM bridge provides spatial separation between rubrene and PtTPTBP to suppress undesirable triplet back transfer while remaining sufficiently thin for efficient FRET-mediated singlet transfer. PtTPTBP was deposited as a thin ∼2 nm film to maintain a continuous sensitizer layer while minimizing the transfer distance to TIPS-ANT, whereas a relatively thick 60 nm TIPS-ANT film provides a large annihilator volume for triplet transport and TTA. These thicknesses represent a functional proof-of-concept architecture rather than a systematic optimization, and further optimization of the individual layers provides an opportunity for future performance improvements.

To fabricate the four-photon upconversion device, a solution of Y6 in chloroform was spin coated onto a fused silica substrate and annealed. The thickness of the Y6 film was approximately 20 nm, and the thicknesses of several spin coated Y6 films were validated using atomic force microscopy (AFM) as shown in Figure S1. The remaining organic films – 10 nm of rubrene, 4 nm of DCM, 2 nm of PtTPTBP, and 60 nm of TIPS-ANT – were subsequently thermally evaporated onto the Y6 film. The final device stack is depicted in Figure 3a. The device was then encapsulated in N_2_ to prevent oxygen quenching of triplets. Under 820 nm NIR excitation, the four-photon upconversion device exhibits strong anti-Stokes emission from both rubrene in the red/yellow region and TIPS-ANT in the blue with a highest-energy emission peak at 445 nm as depicted in Figure 3b. At lower excitation intensity, the blue emission from TIPS-ANT is relatively weak compared to emission from rubrene and DCM. However, as continuous wave (CW) excitation intensity increases from 800 to 5800 mW/cm^2^, the intensity of blue anti-Stokes emission grows rapidly relative to emission from rubrene and DCM. This behavior arises from the compounded nonlinear responses of the two sequential TTA stages. The first TTA stage is pumping the second TTA stage and, therefore, the efficiency of the second stage grows faster than the efficiency of the first stage as excitation intensity increases. As 820 nm produces the strongest anti-Stokes emission, this excitation wavelength is utilized for subsequent power-dependent upconversion. However, the four-photon upconversion device also exhibits anti-Stokes emission under excitation wavelengths as long as 904 nm as shown in Figure 3d. A control device without Y6 showed no anti-Stokes emission under 820 and 904 nm excitation. A control device without the DCM FRET bridge layer showed no blue anti-Stokes emission from TIPS-ANT as shown in Figure S2. By producing blue anti-Stokes emission with the highest-energy emission peak at 445 nm, or 2.79 eV, and an excitation photon energy of 904 nm, or 1.37 eV, this device achieves an anti-Stokes shift of approximately 1.41 eV as depicted in Figure 3c. Notably, this device exhibits an anti-Stokes shift greater than the excitation photon energy. In other words, the emitted photon energy is 2.03× or over double the excitation photon energy. This exceeds the theoretical maximum anti-Stokes shift, or energy gain, of a conventional two-photon TTA system. In a conventional system, two photons are combined to produce one higher-energy photon of an energy up to double the excitation photon energy. Here, four photons are combined, allowing the device to emit a photon of an energy up to four times the excitation photon energy in theory. Therefore, this device constitutes a fundamental advance in TTA upconversion as it establishes an architecture by which anti-Stokes shifts greater than the excitation photon energy can be achieved.

**Figure 3:**
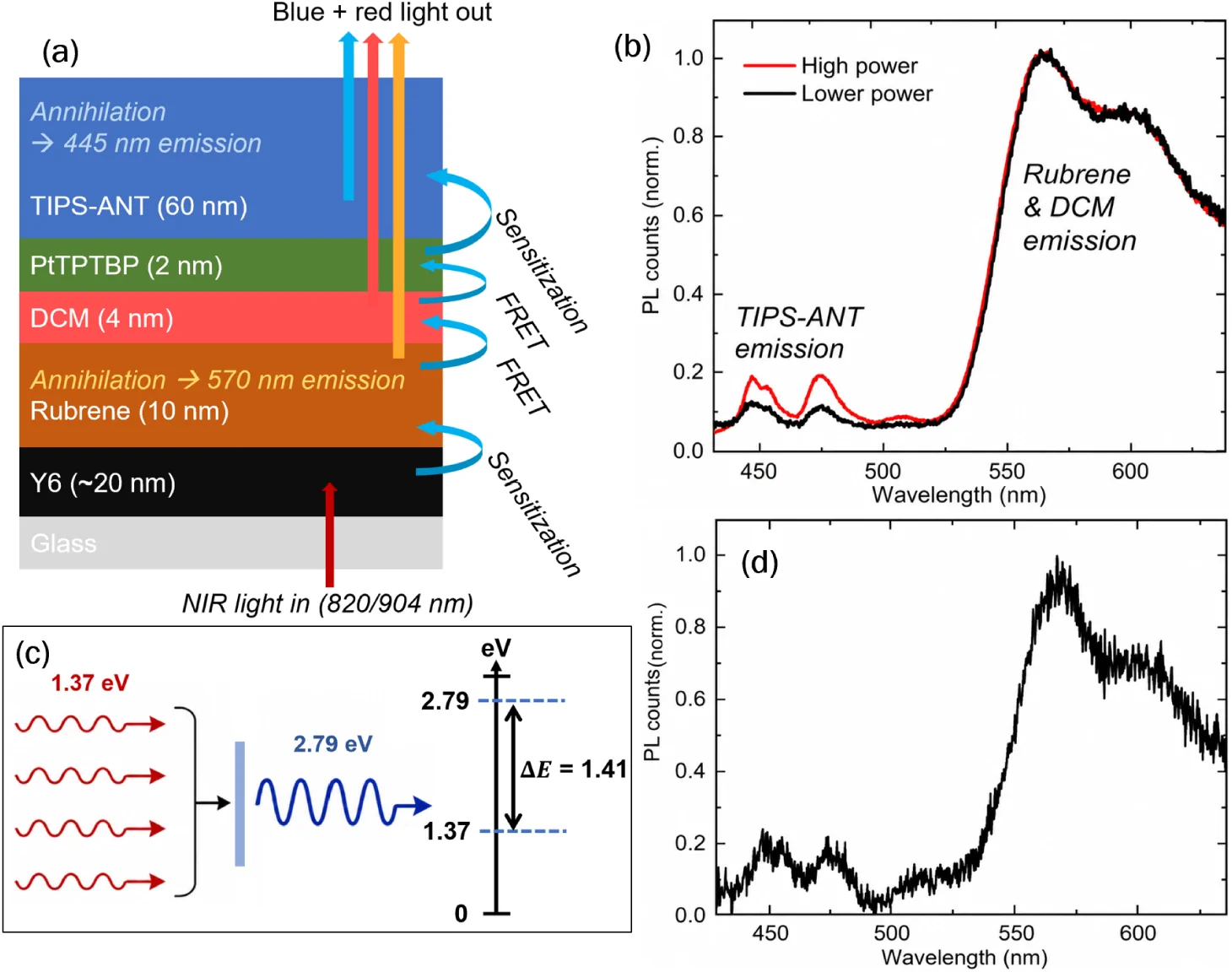
(a) Four-photon upconversion thin-film device structure. (b) Anti-Stokes emission of four-photon upconversion device under 820 nm excitation at lower and higher excitation intensities of 800 and 5800 mW/cm^2^, respectively. (c) Schematic showing four excitation photons being converted into one high-energy photon with achieved anti-Stokes shift relative to the excitation photon energy. (d) Anti-Stokes emission of four-photon upconversion device under 904 nm excitation.

Power-dependent upconversion measurements further support the four-photon nature of the demonstrated upconversion device. At low excitation intensities, a conventional two-photon TTA upconversion process exhibits a quadratic dependence on excitation intensity that switches to a linear dependence at the threshold intensity, *I*_*th*_. The four-photon upconversion device consists of one TTA process pumping a subsequent TTA process. Therefore, at the lowest excitation intensities, the four-photon upconversion should exhibit a quartic dependence on excitation intensity. Then, after one of the constituent TTA processes has crossed *I*_*th*_, the overall power dependence should become quadratic. Then, after the second TTA process has crossed *I*_*th*_ and both processes are above threshold, the overall power dependence should be linear. This progression is summarized in Figure 4d. In practice, as the first TTA process approaches *I*_*th*_, the observed logarithmic slope of the power-dependent upconversion intensity will appear to be between two and four. As the second TTA process approaches *I*_*th*_, the slope will appear to be between one and two.

**Figure 4:**
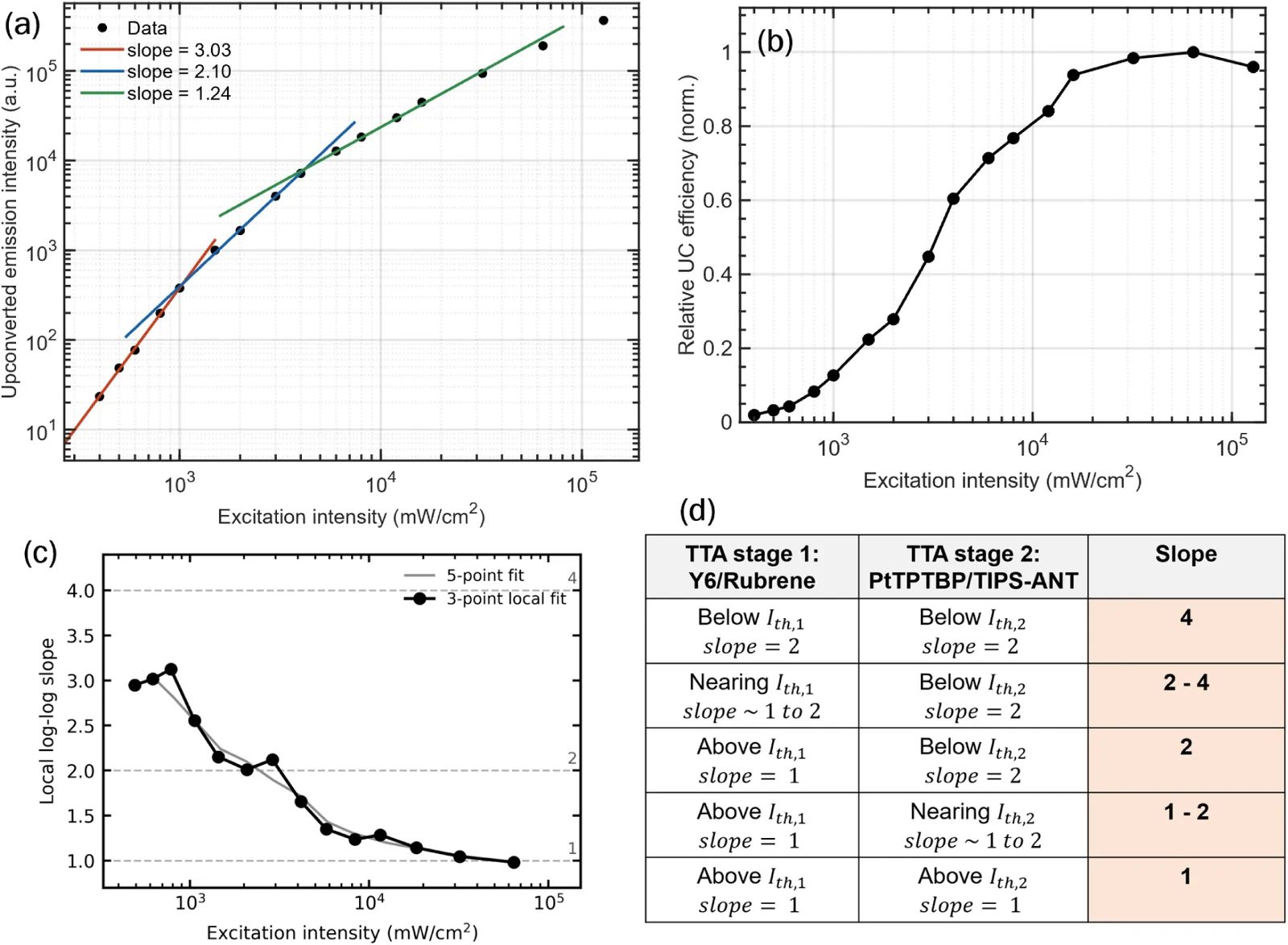
(a) Upconversion emission intensity vs. 820 nm excitation intensity with log-log slope fits for superquadratic, quadratic, and linear power-dependence regimes. (b) Relative upconversion efficiency vs. 820 nm excitation intensity. (c) Local log-log slope analysis of the upconversion intensity vs. excitation intensity plot. (d) Table showing idealized correspondence between below/above threshold operation of each TTA stage and the log-log slope of four-photon upconversion emission on excitation intensity, ranging from quartic to linear.

At the lowest measured excitation intensities, the integrated 445 nm anti-Stokes emission of the four-photon upconversion device exhibits a superquadratic logarithmic slope of 3.03 as depicted in Figure 4a, consistent with a higher-order cascaded upconversion response with the nonlinear output of the first upconversion stage pumping the nonlinear output of the second. Although the ideal low-intensity limit for two sequential quadratic TTA stages would be quartic, the observed slope of 3.03 indicates that one or both stages are already partially approaching their respective TTA thresholds over the experimentally accessible intensity range. As the excitation intensity is increased, the full four-photon device transitions into a quadratic regime with a slope of 2.10 above 981 mW/cm^2^, followed by a transition into a linear regime with a slope of 1.24 above 4170 mW/cm^2^ as shown in Figure 4a. Figure 4c plots the local log-log slope of the data plotted in Figure 4a as a function of the excitation intensity illustrating how the logarithmic slope continuously transitions from superquadratic, to quadratic, to linear, to sublinear as the saturation regime is reached. The relative upconversion efficiency, or the normalized upconversion intensity divided by the excitation intensity, is plotted against the excitation intensity in Figure 4b. Here, the saturation regime can be more clearly seen. At higher excitation intensities, the relative upconversion efficiency exhibits a roll-off commonly observed in solid-state TTA upconversion systems. This behavior is consistent with high intensity saturation and loss processes including triplet-charge annihilation, exciton-exciton annihilation in the annihilator, saturation of interfacial energy transfer, energy back-transfer, and other pathways becoming significant.^6,23^

To establish the behavior of the constituent TTA stages underlying the superquadratic to quadratic to linear response of the complete four-photon device shown in Figure 4, we independently characterized the power-dependent upconversion of the Y6/rubrene (Figure 5a) and PtTPTBP/TIPS-ANT (Figure 5d) bilayers. The independent Y6/rubrene bilayer exhibits anti-Stokes emission from rubrene under 820 nm excitation, as shown in Figure 5b, and shows the characteristic quadratic to linear transition in relative upconversion efficiency as a function of excitation intensity as expected for conventional two-photon TTA, with a threshold intensity of 237.6 mW/cm^2^, as shown in Figure 5c. The threshold intensity for conventional upconversion is commonly designated as the excitation power at which the relative efficiency is 50% of the maximum.^11,16,17^ We assign the first superquadratic to quadratic transition observed in the full four-photon device in Figure 4a at 981 mW/cm^2^ to the Y6/rubrene TTA stage crossing its TTA threshold.

**Figure 5:**
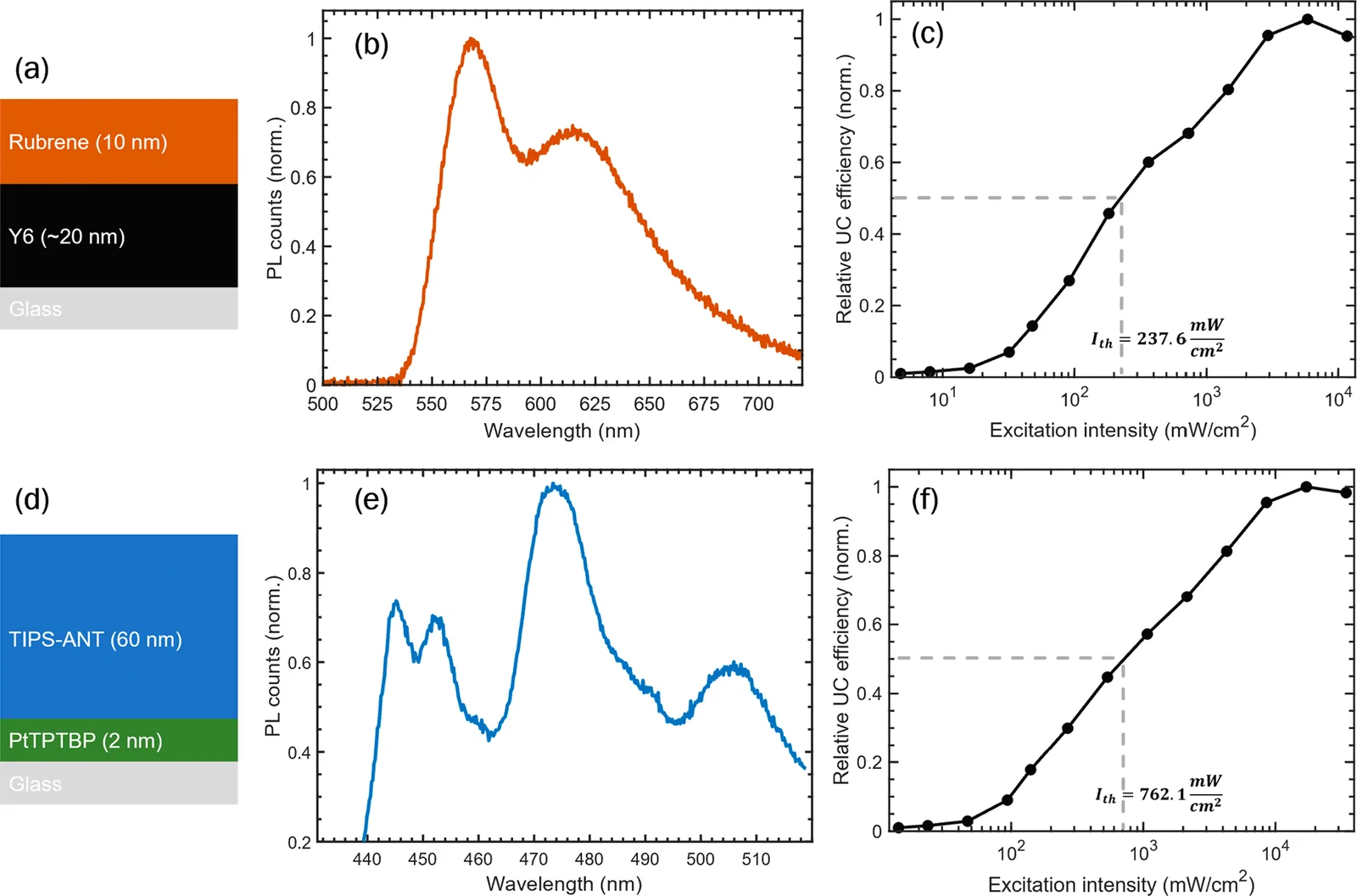
(a) Y6/rubrene TTA bilayer stage. (b) PL of Y6/rubrene TTA stage under 820 nm excitation. (c) Relative upconversion efficiency vs. 820 nm excitation intensity of Y6/rubrene TTA stage showing quadratic and linear regimes and the threshold intensity. (d) PtTPTBP/TIPS-ANT TTA bilayer stage. (e) PL of PtTPTBP/TIPS-ANT TTA stage under 630 nm excitation. (f) Relative upconversion efficiency vs. 630 nm excitation intensity showing quadratic and linear regimes.

The PtTPTBP/TIPS-ANT bilayer also exhibits the expected conventional TTA behavior when measured independently under 630 nm excitation, exhibiting blue anti-Stokes emission from TIPS-ANT, as shown in Figure 5e, and a threshold intensity of 762.1 mW/cm^2^, as shown in Figure 5f.^24^ In the full four-photon device, however, this second TTA stage is not directly excited by far-field 630 nm photons. Instead, it is driven by the internally generated output of the first Y6/rubrene TTA stage via transfer through the DCM FRET bridge. Consequently, the threshold intensity of the independent PtTPTBP/TIPS-ANT bilayer should be interpreted as the direct excitation intensity required to drive the second TTA stage into its efficient linear power-law regime, rather than as the incident 820 nm threshold for the full four-photon device. The full device reaches its second power-law slope transition, from quadratic to linear, at a much higher incident intensity of approximately 4170 mW/cm^2^. This delayed transition is consistent with the second stage being driven indirectly relative to direct optical pumping by the internal energy cascade. However, the quadratic to linear transition of the full four-photon device corresponds to the 820 nm excitation intensity at which the internal pumping of the PtTPTBP/TIPS-ANT stage is driving this second TTA process above threshold in its efficient regime. The measured independent threshold intensities of each stage also suggest that the PtTPTBP/TIPS-ANT bilayer is the bottleneck that controls the onset of the final linear regime. For additional benchmarking of the independent stages, the absolute external quantum efficiency (EQE) was measured for each using an integrating sphere. The Y6/rubrene bilayer exhibits an EQE of approximately 0.03% under 820 nm excitation, while the PtTPTBP/TIPS-ANT bilayer exhibits an EQE of approximately 0.02% under direct 630 nm excitation.

Time-resolved photoluminescence measurements further support the assignment of both bilayers as independent TTA upconversion stages. Under pulsed 820 nm excitation, the Y6/rubrene bilayer exhibits delayed rubrene emission that persists for several microseconds, as shown in Figure 6a. This microsecond-scale delayed fluorescence is consistent with the formation and subsequent bimolecular annihilation of rubrene triplets.^4,25–27^ The approximately 20 nm Y6 layer acts as an effective exciton reservoir that continues to supply rubrene triplets after photoexcitation, as illustrated schematically in Figure 6b.

**Figure 6:**
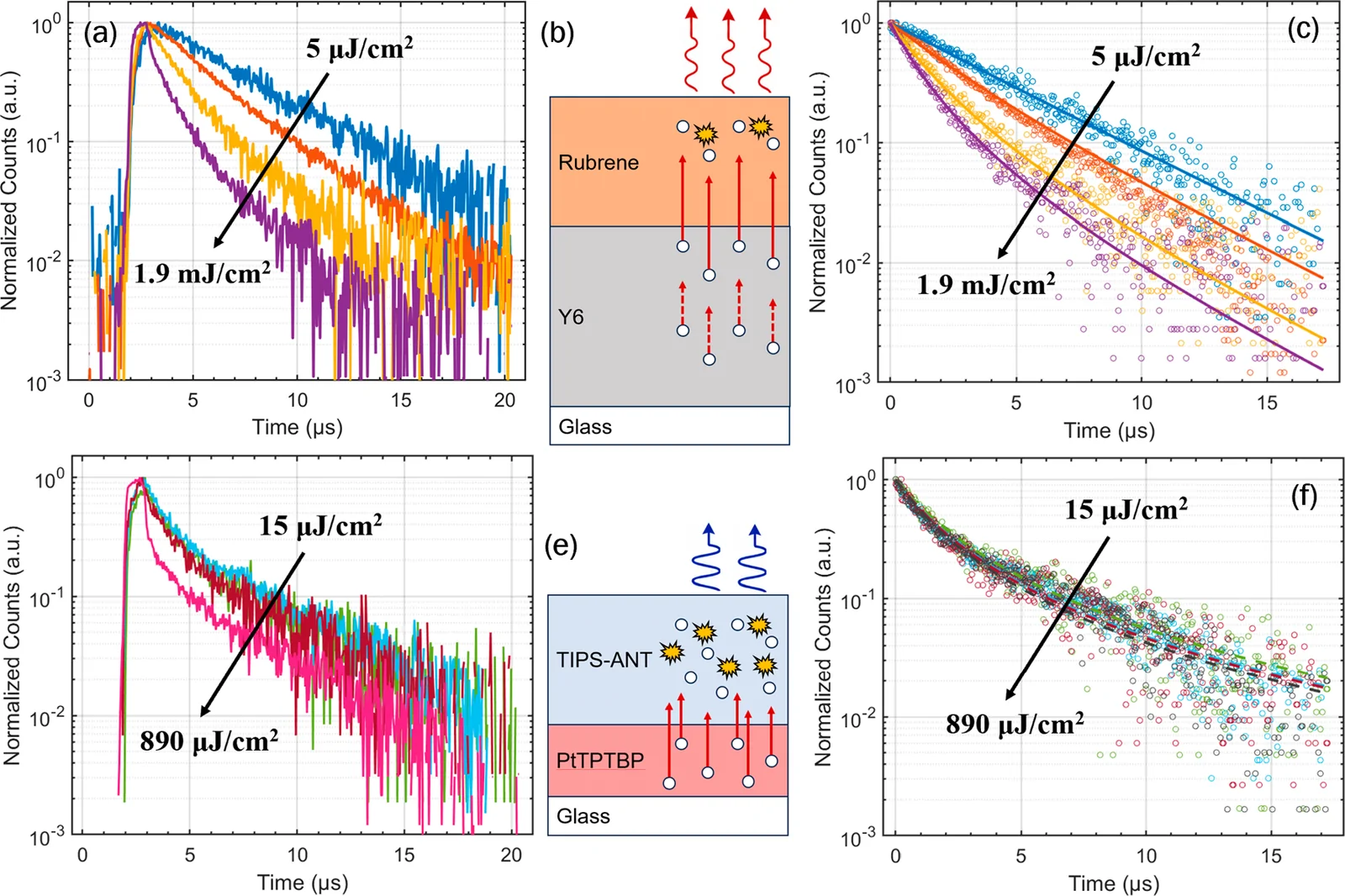
(a) Fluence-dependent normalized TRPL transients of the Y6/rubrene bilayer under pulsed 820 nm excitation. (b) Schematic illustration of the thicker Y6 layer acting as an effective excited-state reservoir that continues to populate rubrene triplets following photoexcitation. (c) Post-peak Y6/rubrene TRPL transient data and fits obtained using the effective coupled rate-equation model. (d) Fluence-dependent normalized TRPL transients of the PtTPTBP/TIPS-ANT bilayer under pulsed 630 nm excitation. (e) Schematic illustration of the thin PtTPTBP layer providing a more temporally confined source of TIPS-ANT triplets. (f) Post-peak PtTPTBP/TIPS-ANT TRPL transient data and corresponding effective rate-equation fits.

At an excitation fluence of 5 μJ/cm^2^, the rubrene emission exhibits a 10–90% rise time of approximately 0.83 μs and a 1/e decay time of approximately 4.5 μs. As the fluence increases to 1.9 mJ/cm^2^, the rise time decreases to approximately 0.45 μs and the decay time decreases to less than 1 μs. The fluence-dependent rise and decay times are summarized in Figure S3 and Figure S4. The increasingly rapid decay at higher fluence is consistent with a growing contribution from bimolecular TTA relative to first-order triplet relaxation. At low triplet density, TTA is comparatively inefficient and the delayed emission persists as the Y6 exciton reservoir continues to populate rubrene triplets. At higher fluence, the larger rubrene triplet population produces more rapid TTA at early times, accelerating both the rise and decay of the delayed fluorescence.

The PtTPTBP/TIPS-ANT bilayer similarly exhibits delayed TIPS-ANT emission under pulsed 630 nm excitation, confirming TTA upconversion in the second bilayer, as shown in Figure 6d. At 15 μJ/cm^2^, the TIPS-ANT emission exhibits a 10–90% rise time of approximately 0.40 μs and a 1/e decay time of approximately 1.5 μs. At 890 μJ/cm^2^, these values decrease only modestly to approximately 0.30 and 0.8 μs, respectively. Thus, the PtTPTBP/TIPS-ANT transients exhibit substantially weaker fluence dependence than the Y6/rubrene transients over the measured range.

This contrast is consistent with differences in exciton transfer from the two sensitizer layers. The 2 nm PtTPTBP layer provides a spatially confined source of triplets and, therefore, transfers excitons over a shorter temporal window, as illustrated in Figure 6e. The TIPS-ANT emission therefore more closely reflects the relaxation and annihilation of an initially generated triplet population. In comparison, the thicker Y6 layer provides a more persistent effective reservoir that continues to populate rubrene triplets after initial excitation.

To quantify these dynamics, the post-peak portions of the TRPL transients were fitted using the simplified rate equation model provided in eqs S1–S3. The model contains the sensitizer exciton density, *T*_*S*_, the annihilator triplet density, *T*_*A*_, excitation transfer from *T*_*S*_ to *T*_*A*_, and bimolecular annihilation of *T*_*A*_ to produce annihilator singlet excitons with rate coefficient, *γ*_TT,A_. Because the natural singlet decay rate is much greater than the annihilation rate, the singlet density is in quasi-steady state and thus the upconverted emission intensity is proportional to the instantaneous TTA singlet generation rate, $I_{\mathrm{UC}}\left(t\right)\propto \gamma_{\mathrm{TT},A}T_{A}^{2}$ . The early-time peak region was excluded as it contains the instrument-response contribution and additional prompt dynamics that are not described by this simplified model. The experimental transients and corresponding fits are shown in Figure 6c and Figure 6f.

For the Y6/rubrene bilayer, the fits yield an effective Y6 source exciton lifetime of approximately 4.5 μs, an effective transfer rate of 2.4 × 10^6^ s^–1^, a rubrene triplet lifetime of approximately 8.6 μs, and an effective TTA coefficient of 4.8 × 10^–15^ cm^3^/s.^28,30^ The rubrene triplet lifetime lies within the range reported for rubrene films, although it is shorter than the tens of μs triplet lifetimes reported for thicker or more crystalline rubrene samples.^27,28,30^ This shorter lifetime is reasonable for the present 10 nm rubrene bilayer where intrinsic triplet decay, interfacial quenching at the Y6/rubrene interface, disorder- or trap-assisted loss, and morphology-dependent triplet transport may result in a shorter lifetime. The rubrene annihilation rate coefficient also falls within the broad range of effective TTA rate coefficients reported for disordered organic thin films.^27,30^ Here, the Y6 source population represents the population responsible for generating rubrene triplets. The model reproduces the strong acceleration of the rubrene emission decay with increasing fluence.

For the PtTPTBP/TIPS-ANT bilayer, the fits yield an effective PtTPTBP source triplet lifetime of approximately 1.6 μs and an effective transfer rate of 1.21 × 10^7^ s^–1^, consistent with more rapid and temporally confined population of the TIPS-ANT layer. Here, we fix the TIPS-ANT triplet lifetime and annihilation rate coefficient to 80 μs and 5 × 10^–12^ cm^3^/s, respectively, based on prior literature reports since our data set does not fully constrain them.^29,30^ These results confirm the role of TTA upconversion in each constituent stage of the full cascaded four-photon upconversion device and are consistent with the threshold intensities for each identified in Figure 5.

The control devices, excitation intensity dependence, and delayed emission dynamics support a cascaded TTA mechanism rather than two-photon absorption as the origin of the blue upconverted emission. Removing Y6 eliminates the anti-Stokes emission, while removing the DCM bridge eliminates the blue TIPS-ANT emission, supporting the involvement of the first sensitizer and interstage FRET coupling. The superquadratic power dependence of the complete device (Figure 4), together with the delayed fluorescence characteristic of TTA in both constituent bilayers (Figure 6), provides additional evidence for two sequential TTA stages. Under 904 nm excitation, the 445 nm emission corresponds to a photon energy of 2.79 eV, exceeding the combined energy of two excitation photons (approximately 2.74 eV), providing additional support for the proposed multistage mechanism.

The low EQE of the present four-photon device arises from the nature of the multistep energy transfer cascade. The individual Y6/rubrene and PtTPTBP/TIPS-ANT TTA stages exhibit EQEs of approximately 0.03% and 0.02%, respectively, whereas the upconverted emission of the complete four-photon device lies below the sensitivity of the integrating sphere measurement, corresponding to an EQE upper bound of <0.001%. In the four-photon architecture, losses associated with NIR absorption and triplet generation in the first stage, TTA in rubrene, sequential singlet transfer through the DCM bridge, triplet generation and transfer in the second stage, TTA in TIPS-ANT, triplet back transfer, and fluorescence and optical outcoupling of the final singlets are compounded. Additional losses may arise from interfacial and trap-assisted exciton quenching and, at higher excitation intensities, triplet-charge and exciton–exciton annihilation. Future optimization should therefore focus on lowering the thresholds and increasing the quantum yields of the independent TTA stages, optimizing the material and thickness of the FRET bridge to maximize forward singlet transfer while suppressing triplet back transfer, reducing interfacial and morphology-dependent quenching, increasing NIR absorption, and improving outcoupling of the final singlets.

## 3 Conclusion

This work demonstrates a fully organic thin-film device architecture for molecular four-photon upconversion by cascading two TTA upconversion stages through an intermediate FRET bridge. In the demonstrated device, NIR excitation is first absorbed by a Y6/rubrene TTA bilayer, producing intermediate upconverted singlets in rubrene. These singlets are then transferred via downhill FRET through a DCM bridge layer to a PtTPTBP/TIPS-ANT bilayer, where a second TTA process generates blue anti-Stokes emission from the TIPS-ANT annihilator. This sequential energy transfer cascade converts 904 nm excitation photons into 445 nm emission, corresponding to an emitted photon energy of 2.79 eV, an anti-Stokes shift of 1.41 eV, and an emitted photon energy that is 2.03× the excitation photon energy. Because this anti-Stokes shift is larger than the excitation photon energy itself, the demonstrated device exceeds the maximum photon energy gain accessible to a conventional two-photon TTA upconversion system.

Beyond extending the possible anti-Stokes shift, this cascaded architecture also introduces a higher-order nonlinear optical response that is not available in conventional TTA upconversion. Since the nonlinear output of the first TTA stage serves as the effective pump for the second nonlinear TTA stage, the ideal low-intensity response of the full device is quartic when both stages are below their respective thresholds. Experimentally, the device exhibits a superquadratic power-law slope of 3.03 at the lowest measured excitation intensities, followed by transitions to quadratic and linear regimes as the constituent TTA stages cross their thresholds. Power-dependent measurements of the individual Y6/rubrene and PtTPTBP/TIPS-ANT bilayers, together with fluence-dependent TRPL measurements, confirm that both bilayers operate as independent TTA upconversion stages and support the assignment of the observed nonlinear response to a cascaded four-photon mechanism.

Therefore, this work establishes a new design principle for TTA upconversion thin films: coupling multiple nonlinear TTA stages can overcome the two-photon energy-gain limit of conventional TTA while simultaneously producing steeper optical nonlinearity. In principle, an ideal four-photon upconversion system could generate emitted photon energies up to four times the excitation photon energy, corresponding to anti-Stokes shifts far beyond those accessible with a single TTA stage. Such molecular upconversion responses could be useful for photoredox catalysis, photochemistry, optical thresholding, high-contrast imaging, nonlinear sensing, and other applications where steep intensity-dependent emission and/or large anti-Stokes shifts are desirable.

The present device should be viewed as a proof-of-concept demonstration rather than an optimized platform. The blue upconverted emission of the full device exhibited an EQE below the measurement sensitivity and exhibited relatively high excitation thresholds. As discussed, this low efficiency reflects compounding losses across the multistage energy cascade. Further optimization of the constituent TTA stages, FRET bridge, interfaces, layer thicknesses, and optical architecture should provide routes toward substantially improved efficiency and thresholds while retaining the large anti-Stokes shift and higher-order nonlinear response demonstrated here.

## 4 Experimental Section

### 4.1 Sample Fabrication

To spin coat Y6 films, a solution of Y6 in chloroform was fabricated at 3 mg/mL. Spin coating was performed by dropping 150 μL of the solution onto the fused silica substrate and spinning at 500 rpm for 5 s, followed by 3000 rpm for 60 s. The spin coated films were then annealed on a hot plate at 75 °C for 8 min. This spin coating procedure was optimized to fabricate Y6 films with a thickness of ∼20 nm. All organic films, with the exception of Y6, were fabricated via physical vapor deposition (PVD) using an Angstrom high vacuum thermal evaporator with a base pressure of ∼1 × 10^–7^ Torr. The 10 nm rubrene layer was evaporated onto the Y6 film at 0.5 Å/s, followed by 4 nm of DCM at 0.1 Å/s, 2 nm of PtTPTBP at <0.1 Å/s, and 60 nm of TIPS-ANT at 2 Å/s. TIPS-ANT was synthesized according to a previously reported procedure.^31^ The spectroscopic properties of PtTPTBP were identical to those previously reported.^32^ Glass substrates were sonicated in acetone and IPA and treated with O_2_ plasma immediately before deposition. Samples were encapsulated in a dry N_2_ glovebox using a UV-cured polymer to prevent O_2_ quenching of triplets.

### 4.2 Experimental Measurements

The optical power density for excitation with coherent laser diode sources was measured with a photodiode and divided by the 1/e^2^ area of the beam as captured by a CMOS camera as shown in Figure S5. The intensity of anti-Stokes emission was calculated by integrating over the anti-Stokes emission spectra. The relative upconversion efficiency is calculated by dividing the anti-Stokes emission intensity by the respective optical power density. The EQE was measured using an integrating sphere to calculate the total number of upconverted photons emitted by the sample – obtained by integrating the anti-Stokes emission spectrum – divided by the total number of incident excitation photons on the sample – obtained by integrating the excitation laser spectrum incident on a blank sample. AFM images were acquired using a Veeco NanoScope IV system with a Digital Instruments Dimension 3100 in tapping mode, using Bruker RTESPA-150 and RTESPA-300 probes. For TRPL measurements, samples were excited using a Light Conversion PHAROS2-10 femtosecond laser coupled to a Light Conversion ORPHEUS-HE collinear optical parametric amplifier. The laser was operated at a repetition rate of 10 kHz with a pulse width of 290 fs, and intensity was controlled using ND filters. The laser spot size was determined using the FWHM of the spot as captured by a CMOS camera. Emission was collected using a Teledyne Princeton Instruments SP-2150i monochromator coupled to a Hamamatsu C10910 streak camera as shown in Figure S6.

## Supplementary Material



## Data Availability

The data that support the findings of this study are available from the corresponding author upon reasonable request.

## Notes

Additional experimental details and methods including AFM images, a control DCM-free spectrum, TRPL rise and decay times, and fitted rate equations (PDF)
