# Supplementary material for: Four-Photon Molecular Upconversion
Source: J Am Chem Soc. 2026 Oct 2;148(40):43952–61. doi: 10.1021/jacs.6c17238 (PMC13674276; doi:10.1021/jacs.6c17238)
Supplement: Supplementary file 1 [file ja6c17238_si_001.pdf]

Four-Photon Molecular Upconversion

Seamus S. Lowe[a], Maxwell Sun[b], Jesse A. Wisch[c], Kostubh Gaur[d], Chiao-Jung Su[c], Azka Arshad[e], Felix N. Castellano[e], John E. Anthony[d], Noel C. Giebink[b], Barry P. Rand[c, f]*

**Corresponding author*

[a] S.S. Lowe

Department of Mechanical & Aerospace Engineering

Princeton University

Princeton, NJ 08544, USA

[b] M. Sun, Prof. N.C. Giebink

Department of Electrical Engineering and Computer Science

University of Michigan

Ann Arbor, MI 48109, USA

[c] Prof. B.P. Rand, J.A. Wisch, C.J. Su

Department of Electrical and Computer Engineering

Princeton University

Princeton, NJ 08544, USA

E-mail: brand@princeton.edu

[d] Prof. J.E. Anthony, K. Gaur

Department of Chemistry

University of Kentucky

Lexington, KY 40506, USA

[e] Prof. F.N. Castellano, A. Arshad

Department of Chemistry

North Carolina State University

Raleigh, NC 27695, USA

[f] Prof. B.P. Rand

Andlinger Center for Energy and the Environment

Princeton University

Princeton, NJ 08544, USA

Table of Contents

1. Atomic Force Microscopy (AFM) of Y6 films……3

2. No DCM Film Control Spectrum……4

3. TRPL Rise Time for Independent TTA Stages……4

4. Fluence-Dependent Apparent TRPL 1/e Decay Times……5

5. Coupled Rate Equations……6

6. Beam Size……8

7. Raw TRPL Maps……9

1. Atomic Force Microscopy (AFM) of Y6 films

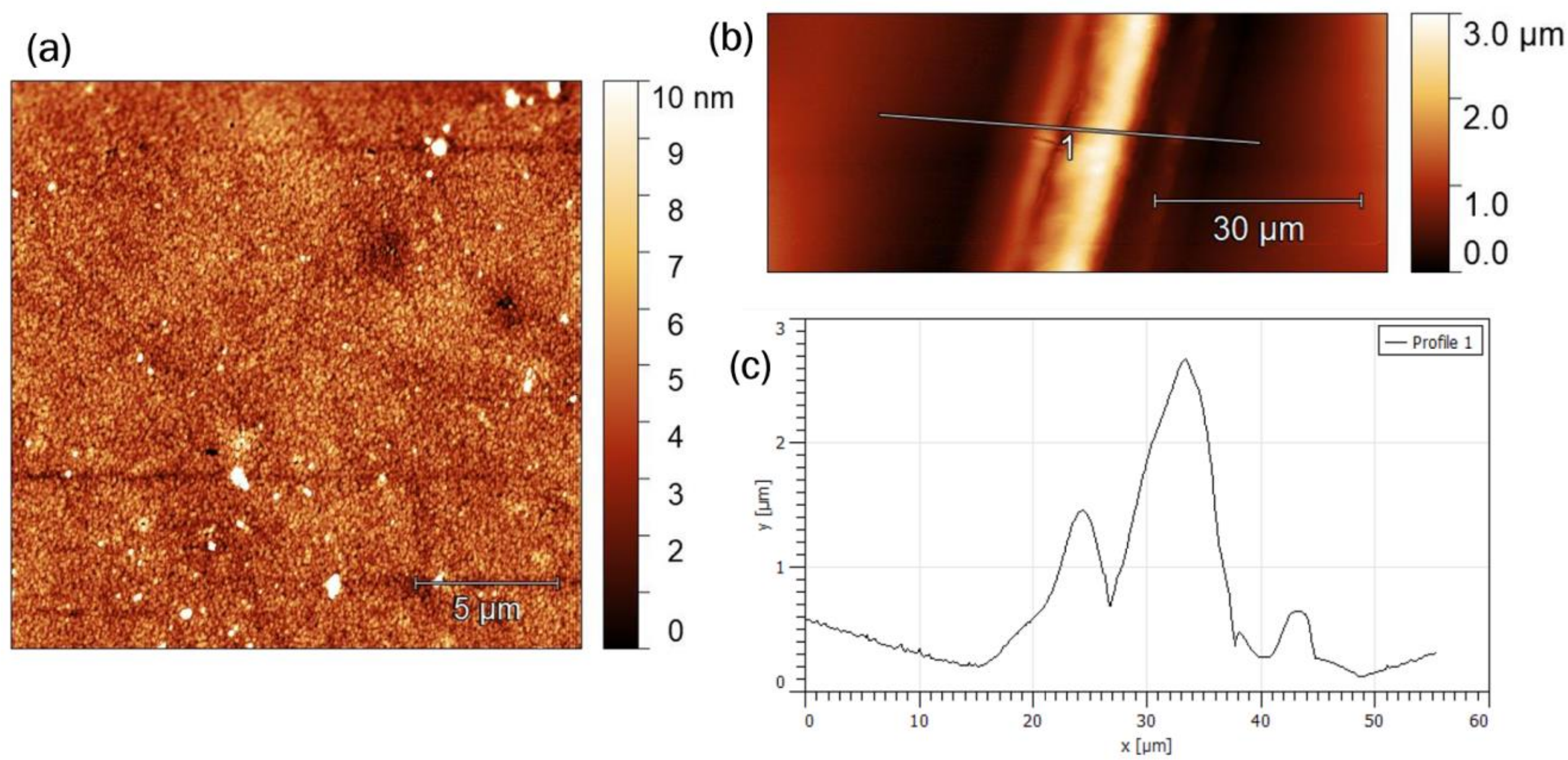

Figure S1: (a) AFM image of Y6 film showing RMS roughness of 1.747 nm. (b) AFM image of coffee ring feature at the edge of Y6 film with Y6 film to the left side of the coffee ring and bare substrate to the right. (c) AFM line profile of line shown in Figure S1b.

Figure S1a shows an AFM image of a Y6 film spin coated on a fused silica substrate with parameters described in the experimental section of the main manuscript. The RMS roughness is calculated to 1.747 nm from the image, which demonstrates that the ~20 nm thick film is continuous and relatively smooth over the inspected region. **Figure S1b** shows an AFM image of the edge of the same Y6 film. At the edge, a coffee ring effect is observed, resulting in a tall peak where material has built up at the film edge. By extracting a line profile along the white line shown in the image, we can obtain the height difference between the Y6 film on the left side of the coffee ring effect, and the bare fused silica substrate surface on the right side of the coffee ring effect. **Figure S1c** shows the line profile of the white line in **Figure S1b**, showing the Y6 film height before the coffee ring effect, the tall ~2.5 µm coffee ring effect, and the bare substrate

height to the right of the coffee ring feature. From the line profile, the Y6 film thickness is estimated to be roughly 18.81$\pm$0.234 nm with a mean squared error (MSE) of 5.5. Therefore, the thickness of the spin coated Y6 films may be estimated as ~20 nm.

2. No DCM Film Control Spectrum

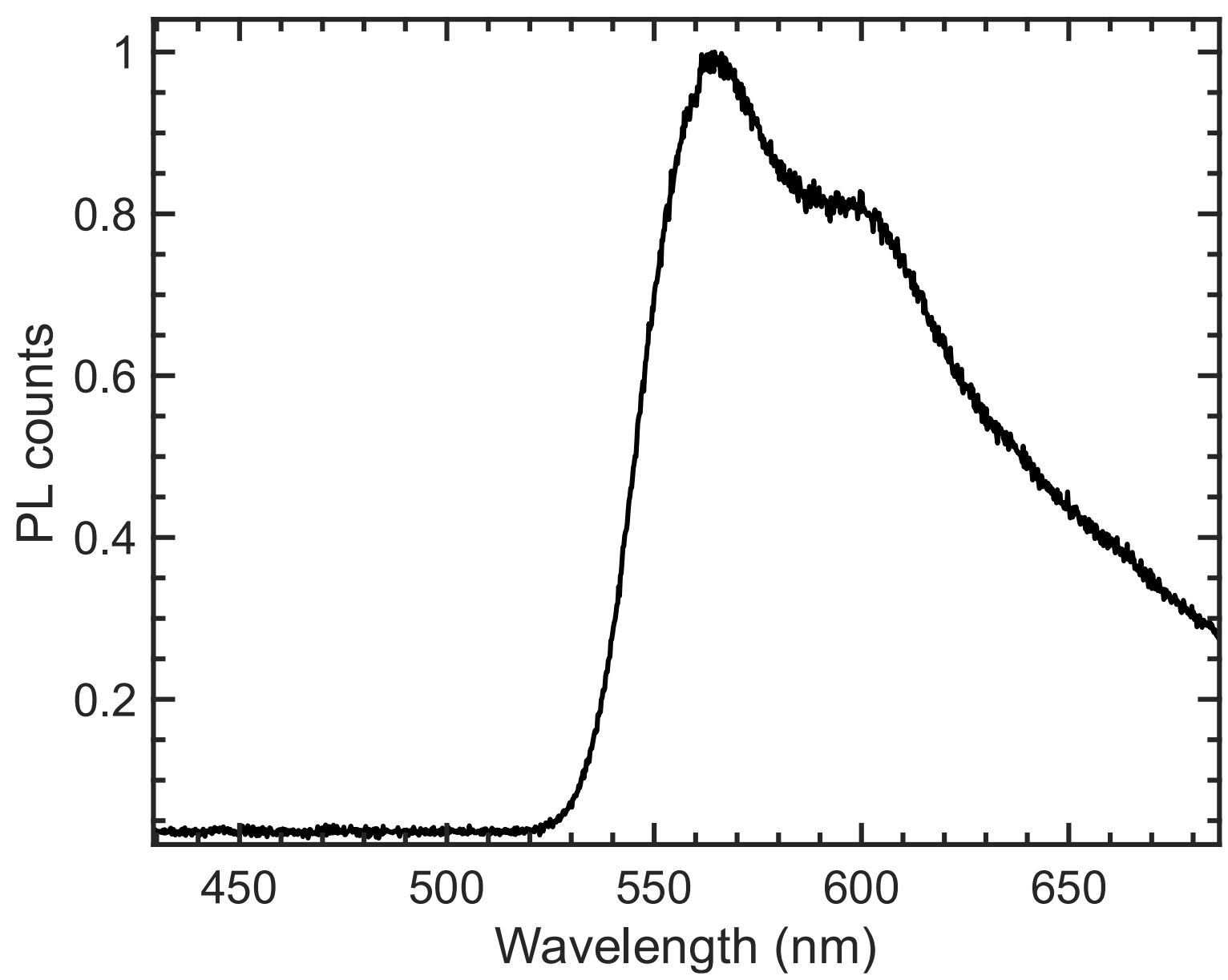

Figure S2: Photoluminescence (PL) spectrum of control device without a DCM film under 820 nm excitation.

Figure S2 shows the PL spectrum of the four-photon upconversion device described in the main manuscript without the DCM FRET bridge film under 820 nm continuous wave excitation. There is no discernable blue anti-Stokes emission from TIPS-ANT while the emission from the first TTA stage where rubrene is the annihilator is strong and spectrally very similar to the residual rubrene emission from the complete four-photon device as depicted in **Figure 3**. Therefore, the

DCM film is necessary to enable the four-photon upconversion energy cascade resulting in blue ant-Stokes emission upon NIR excitation.

3. TRPL Rise Time for Independent TTA Stages

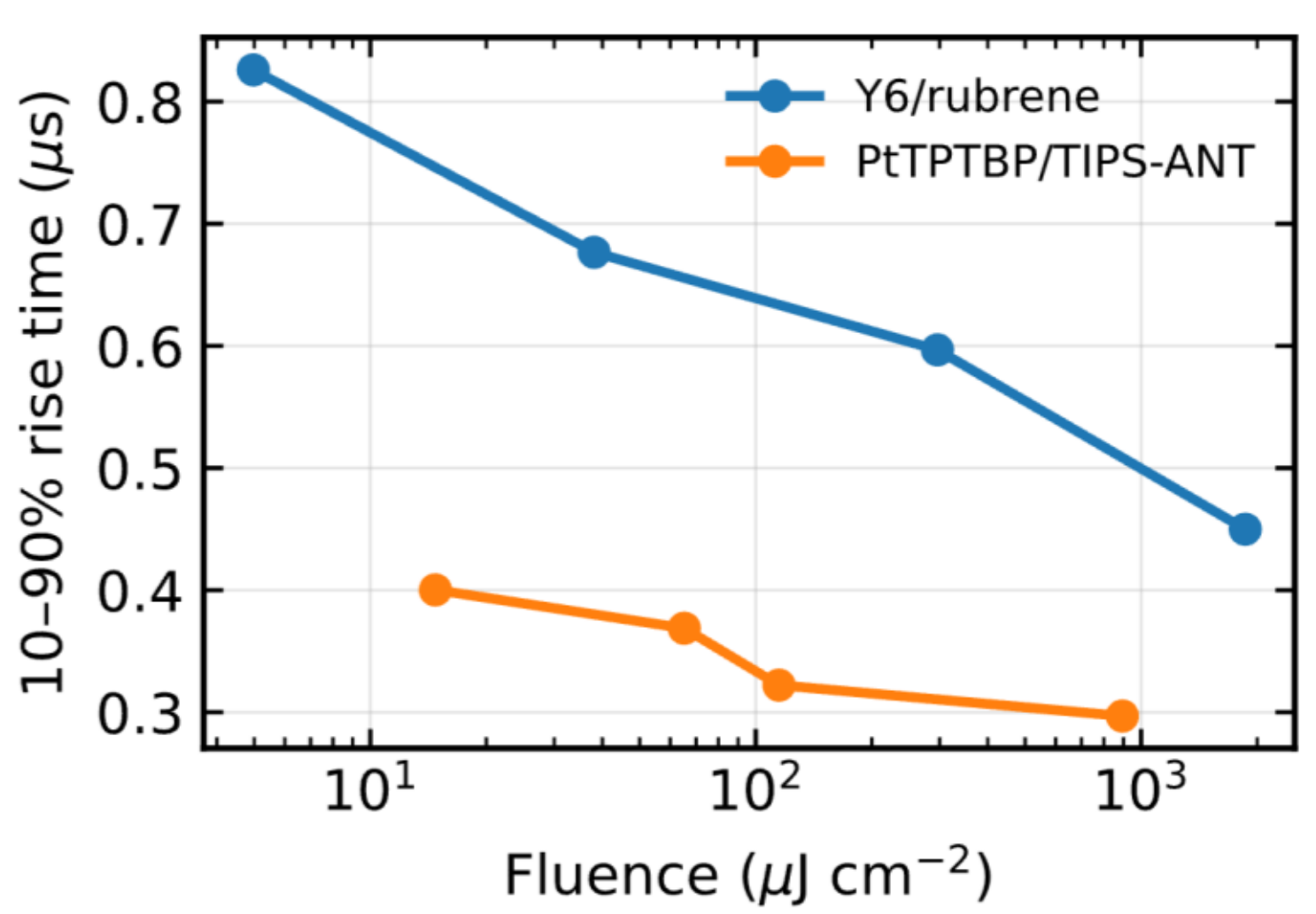

Figure S3: 10-90% rise time of anti-Stokes emission as a function of excitation fluence for Y6/rubrene and PtTPTBP/TIPS-ANT TTA upconversion stages.

Figure S3 plots the 10-90% rise times of the independent Y6/rubrene and PtTPTBP/TIPS-ANT TTA upconversion stages as a function of the excitation fluence. The Y6/rubrene stage exhibits a much more significant fluence-dependence than the PtTPTBP/TIPS-ANT stage, dropping from approximately 0.83 μs at low fluence to 0.45 μs at high fluence. In contrast, the PtTPTBP/TIPS-ANT rise time drops more modestly from 0.4 μs at low fluence to 0.3 μs at high fluence. The rise time of the PtTPTBP/TIPS-ANT stage is much lower than for Y6/rubrene at all fluences, though the difference is more pronounced at lower fluence excitation.

4. Fluence-Dependent Apparent TRPL 1/e Decay Times

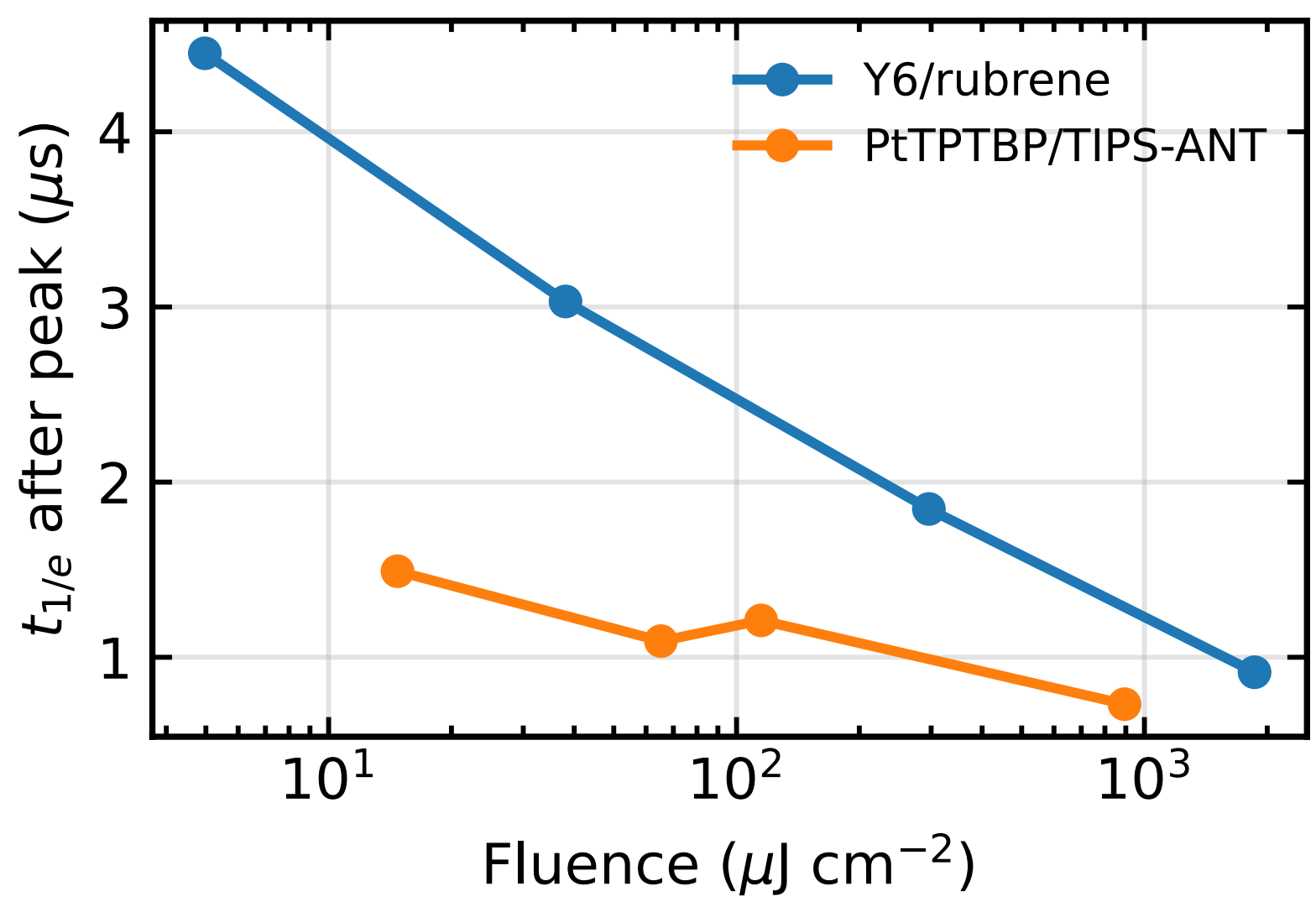

Figure S4: Apparent 1/e decay time of the normalized anti-Stokes TRPL emission from the Y6/rubrene bilayer under 820 nm excitation and the PtTPTBP/TIPS-ANT bilayer under 630 nm excitation as a function of incident excitation fluence.

Figure S4 summarizes the fluence dependence of the apparent 1/e emission decay times for the independent Y6/rubrene and PtTPTBP/TIPS-ANT TTA upconversion stages. For Y6/rubrene, the 1/e decay time decreases strongly from approximately 4.5 µs at an excitation fluence of 5 µJ/cm^2 to less than 1 µs at 1.9 mJ/cm^2. This pronounced shortening is consistent with the increasing contribution of bimolecular TTA at higher rubrene triplet densities. At low fluence, first-order triplet relaxation and continued population of rubrene triplets by the Y6-derived source state allow the delayed emission to persist for several microseconds. At higher fluence, TTA becomes efficient at earlier times and more rapidly depletes the rubrene triplet population.

The PtTPTBP/TIPS-ANT bilayer exhibits a weaker fluence dependence. Its apparent 1/e decay time decreases from approximately 1.5 µs at 15 µJ/cm^2 to approximately 0.8 µs at 890 µJ/cm^2.

This comparatively modest change is consistent with the thin PtTPTBP layer providing a more temporally confined source of TIPS-ANT triplets and with the measured transients spanning a narrower range of TTA kinetic regimes.

5. Coupled Rate Equations

$$\frac{dT_S}{dt} = -\frac{1}{\tau_{T,S}}T_S - k_{TET}T_S, \tag{S1a}$$

$$\frac{dT_A}{dt} = k_{TET}T_S - \frac{1}{\tau_{T,A}}T_A - \gamma_{TT,A}{T_A}^2, \tag{S1b}$$

$$\frac{dS_A}{dt} = \phi\gamma_{TT,A}{T_A}^2 - \frac{1}{\tau_{S,A}}S_A. \tag{S1c}$$

The transient photoluminescence data are modeled using the simplified rate equation model in Equations (S1a), (S1b), and (S1c), which describe the triplet exciton density of the sensitizer, the triplet density of the annihilator, and the singlet density of the annihilator, respectively. Here, a fraction of the initial optically-excited singlet density in each sensitizer material (Y6 or PtTPTBP) is assumed to rapidly produce triplet states (via recombination of donor-acceptor charge transfer states or intersystem crossing, respectively) with natural lifetime, $\tau_{T,S}$. These triplets transfer to the annihilator (rubrene or TIPS-ANT) with an effective triplet transfer rate, k_{TET}, and subsequently decay with natural lifetime, $\tau_{T,A}$, or annihilate with bimolecular rate $\gamma_{TT,A}$. A fraction of the annihilation events (ϕ) produce annihilator singlet excitons with density $S_A(t)$ and natural lifetime $\tau_{S,A}$. Because the annihilator singlet lifetime is much shorter than the microsecond-scale triplet dynamics, the annihilator singlet density is in quasi-steady state and thus the upconverted luminescence intensity, I_{UC}, is proportional to the triplet-triplet annihilation rate:

$$I_{\mathrm{UC}}(t) \propto \frac{S_A}{\tau_{S,A}} \approx \phi\gamma_{\mathrm{TT},A}T_A^2. \tag{S2}$$

Equations (S1) and (S2) are solved numerically using an initial condition for T_S based on the absorbed pulse fluence, and each set of fluence-dependent TRPL data is globally fit for k_{TET}, $\tau_{T,S}$, $\tau_{T,A}$, and $\gamma_{\mathrm{TT},A}$. The results are provided in Table S1 below. The values for $\tau_{T,A}$ and $\gamma_{\mathrm{TT},A}$ in the case of Y6/rubrene are in reasonable agreement with those measured previously in Ref. X. Note that in the case of PtTPTBP/TIPS-ANT, $\tau_{T,A}$ and $\gamma_{\mathrm{TT},A}$ are held fixed as they are poorly constrained due to the minimal exciton density-dependence observed in the transients.

Table S1. Fit results for transient photoluminescence data

**Parameter**	**Y6/rubrene**	**PtTPTBP/TIPS-ANT**
k_{TET} (s^{-1})	2.4×10^6	1.5×10^7
$\tau_{T,S}$ (μs)	4.5	1.4
$\tau_{T,A}$ (μs)	8.6	35
$\gamma_{\mathrm{TT},A}$ (cm^3 s^{-1})	4.8×10^{-15}	1×10^{-12}

Adding a generation term to Eqn. (S1a) and solving the system in steady state, it is straightforward to show that the threshold generation rate at which the upconverted emission transitions from quadratic to linear scaling is:

$$\mathrm{G} = \frac{1}{4}\left(1 + \frac{1}{\tau_{T,S}k_{\mathrm{TET}}}\right)\left(\frac{1}{\gamma_{\mathrm{TT},A}{\tau_{T,A}}^2}\right). \tag{S3}$$

The generation rate in turn is related to the incident light intensity via $\mathrm{G} \approx \phi_{\mathrm{abs}}\eta \mathrm{I}_0/(dh\nu)$, where ϕ_{abs} is the fraction of light absorbed by the sensitizer, d is its thickness, $h\nu$ is the incident photon energy, and η is the yield of sensitizer triplet excitons per absorbed photon. Plugging in

the values in **Table S1** and estimating $\phi_{\mathrm{abs}} \approx \alpha d$ from the absorption coefficient of the sensitizer, the threshold intensity obtained for Y6/rubrene in Fig. 5 yields $\eta \sim 3.2$, which is on the order of unity and thus indicates that triplet production in this system is highly efficient. In the PtTPTBP/TIPS-ANT system, the parameters in **Table S1** yield $\eta \sim 6.4 \times 10^{-4}$. This low value may reflect the existence of TTA within the PtTPTBP sensitizer, which is not accounted for in the model, but could suppress the triplet density making η appear lower than it actually is.

6. Beam Size

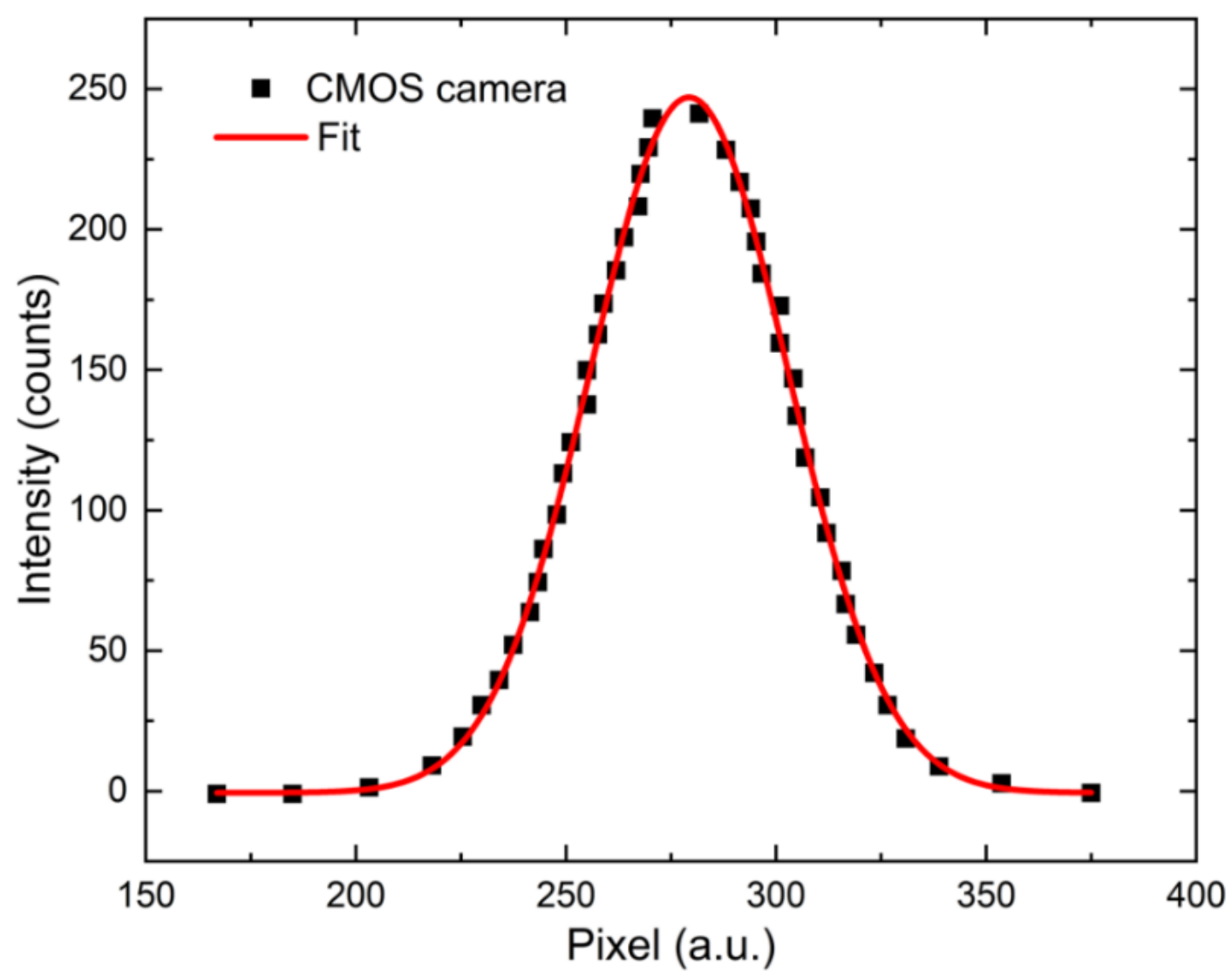

Figure S5: Gaussian fit to beam profile points from CMOS beam capture.

Beam size was measured using a Thorlabs DCC1645C CMOS camera and fit to a Gaussian in horizontal and vertical directions. The $1/e^2$ method was used to calculate the horizontal and vertical beam diameters. The beam area is calculated from these diameters. An example of this is shown in **Figure S5** above.

7. Raw TRPL Maps

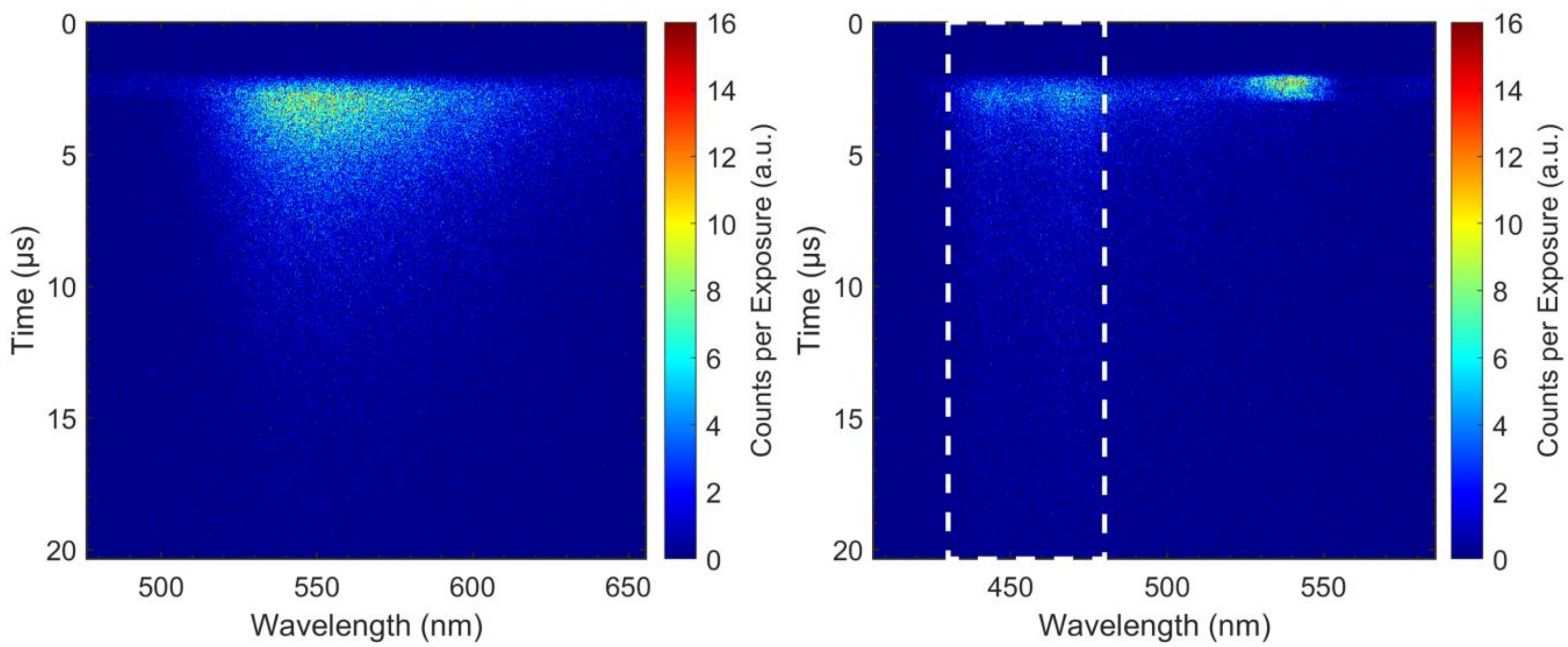

Figure S6: (left) Raw TRPL map of Y6/rubrene bilayer under 820 nm excitation. (right) Raw TRPL map of PtTPTBP/TIPS-ANT under 630 nm excitation.

The raw TRPL map of the Y6/rubrene bilayer is shown in **Figure S5 left** shows the strong anti-Stokes emission from rubrene which decays as a function of time after excitation with the emission peak near 550 nm. **Figure S5 right** shows the same for the PtTPTBP/TIPS-ANT under 630 nm excitation with two anti-Stokes emission peaks from TIPS-ANT – the highest energy peak being observed at 445 nm. The prompt emission at approximately 545 nm in Fig. S5 right may correspond to weak PtTPTBP singlet emission that results from TTA in the sensitizer, which is expected to be significant at the fs pulse fluences used in the TRPL measurements.(2) The kinetic traces from the full spectral range of rubrene and boxed TIPS-ANT region in the TRPL maps are analyzed in **Figure 6** of the main manuscript.

References

(1) Ryasnyanskiy, A.; Biaggio, I. Triplet Exciton Dynamics in Rubrene Single Crystals. Phys. Rev. B **2011**, 84, 193203.

(2) Kéna-Cohen, S.; Forrest, S. R. Green Polariton Photoluminescence Using the Red-Emitting Phosphor PtOEP. Phys. Rev. B **2007**, 76, 075202.
